# Mechanisms Influencing Ferromagnetic Resonance Linewidth in Ca–In–Sn Co-Substituted Yttrium–Iron Garnet Ferrites

**DOI:** 10.3390/ma18235331

**Published:** 2025-11-26

**Authors:** Yiwei Hu, Xiansong Liu, Shuangjiu Feng

**Affiliations:** Engineering Technology Research Center of Magnetic Materials of Anhui Province, School of Materials Science and Engineering, Anhui University, Hefei 230601, China

**Keywords:** sintering, porosity, magnetic properties, soft magnets

## Abstract

With the rapid development of communication technologies such as 5G, yttrium iron garnet (YIG) has been widely applied in microwave devices and other systems owing to its low ferromagnetic resonance linewidth. Loss reduction and effects of doping on performance have been important research areas for garnet ferrite. This study prepared Ca^2+^, In^3+^, and Sn^4+^ codoped YIG ferrite samples with the chemical formula Y_3−*x*_Ca*_x_*Fe_5−*x*−*y*_In*_y_*Sn*_x_*O_12_ (*x* = 0.05–0.3) (*y* = 0.2, 0.45) via solid-state reaction. The analyses of the crystal structure, micromorphology, and magnetic properties enabled the identification of the causes of variations in parameters, such as saturation magnetization and coercivity. Theoretical calculations of the anisotropy constants clarified the patterns upon substituting Fe^3+^ with In^3+^ and Sn^4+^, revealing a shift in the positions of Fe^3+^ substitution. Finally, the primary factors influencing loss were identified, and the key process parameters influencing performance were determined. The resulting polycrystalline garnet ferrite exhibited an extremely low ferromagnetic resonance linewidth parameter (ΔH = 29 Oe) and a high density (>5.2 g/cm^3^). This study provides specific guidance on process parameters and element selection for high-performance, low-loss YIG materials, as well as a detailed theoretical explanation of the performance changes resulting from co-doping YIG with In^3+^ and Sn^4+^.

## 1. Introduction

Yttrium iron garnet (Y_3_Fe_5_O_12_, YIG) has a small anisotropy constant and narrow ferromagnetic resonance linewidth, and electromagnetic waves can be transmitted and controlled with low loss in this material [1]. Therefore, it is widely used in devices for transmitting, receiving, and filtering microwave signals. The market for YIG-based devices is large, and includes wireless base stations, the automotive industry, aerospace, and other fields [2,3]. YIG has a body-centered cubic space group, Ia3d, featuring three types of cation sites: dodecahedral (c sites), tetrahedral (d sites), and octahedral (a sites). Each cell contains 24 c, 24 d, and 16 a sites for a total of 64 interstitial positions occupied by metal ions [4]. In yttrium–iron garnet (YIG) crystals, Y^3+^ and Fe^3+^ ions occupy the c- and d-sites, respectively, while the ratio of the Fe^3+^ at the d-sites to that at the a-sites is 3:2. Notably, the magnetic moment of YIG stems from the antiparallel arrangement of Fe^3+^ ions at the a- and d-sites [5].

Dastjerdi et al. studied the lattice distortion caused by the co-replacement of Ce, La, and Pr (rare earth) ions in YIG and the effect of these substitutions on the superexchange interaction of Fe^3+^ ions in the tetrahedral (d) and octahedral (a) positions [6]. Niyifar et al. synthesized a Y_3_In*_x_*Fe_5−*x*_O_12_ ferrite material via a solid-phase method and found that In^3+^ addition caused the lattice constant to increase, and the saturation magnetization first decreased and then increased with increasing In^3+^ content [7]. Bouziane et al. found that when Cr^3+^ and Al^3+^ are co-doped in YIG, the preferential tendency of the ion substitution sites remains essentially the same as when YIG is doped with either Cr^3+^ or Al^3+^ alone [8]. Yang et al. found that Ca^2+^ and Sn^4+^ increased the saturation magnetization of the sample. A Ca^2+^ and Sn^4+^ substitution amount of 0.3 resulted in high performance: M_s_ = 2073 Gs and ΔH = 42 Oe [9]. Cao et al. synthesized Y_3_Zn*_x_*Zr*_x_*Fe_5−2*x*_O_12_ and reported that replacing Fe^3+^ with Zr^4+^ at the octahedral a-sites reduced the magnetic permeability, magnetic loss, and saturation magnetization of the material [10]. The introduction of In^3+^ into YIG can reduce its anisotropy constant [7,11], while the simultaneous introduction of In^3+^ and Sn^4+^ can change its magnetic properties [12]. Notwithstanding these studies, research on the magnetic properties of In^3+^ and Sn^4+^ ions co-substituting for Y^3+^ in YIG, and on the ferromagnetic resonance linewidth theory, remains incomplete. There is still room to improve YIG performance (loss reduction) through these elements. Furthermore, process improvements for YIG and reductions in sintering costs can be realized through optimizing the chemical composition of the material.

Achieving a small/narrow ferromagnetic resonance linewidth (ΔH) in garnet ferrites is crucial for communication equipment. In this study, we present an optimized synthesis route for a novel garnet, Y_3−*x*_Ca*_x_*Fe_5−*x*−*y*_In*_y_*Sn*_x_*O_12_. This material exhibits very low ferromagnetic resonance loss and high density, achieved with reduced sintering temperatures and low production complexity. This material offers a promising pathway for advancing research on garnet ferrites. Detailed theoretical calculations explain the saturation magnetization and anisotropy constant and identify the primary factors influencing the loss. Several complementary experimental methods were employed to comprehensively analyze the variations and trends in the Ca–In–Sn ternary ion occupancy. The proposed research approach demonstrates considerable advantages, particularly the comprehensive theoretical treatment of microwave loss mechanisms, which is more extensive than previous studies. This paper not only proposes a solution for low-loss YIG but also provides a comprehensive performance-optimization strategy, enabling improved performance of this material.

## 2. Materials and Methods

### 2.1. Sample Preparation

In this study, we used Y_2_O_3_ (99.9% purity), Fe_2_O_3_ (99.9% purity), CaCO_3_ (99.9% purity), In_2_O_3_ (99.99% purity), and SnO_2_ (99.9% purity) as the raw oxide materials. All raw materials were purchased from Macklin, Shanghai, China. Each raw material was weighed according to its chemical formula mass ratio. The amount of Fe_2_O_3_ was reduced by 1% of its calculated mass to compensate for the additional Fe produced by steel balls and steel cans during ball milling [13]. The raw materials were mixed in the following proportions: M_material_:M_ball_:M_water_ = 1:4.2:1.4; they were ground by a planetary ball mill at 230 rpm for 6 h. After ball milling, the mixture was dried at 90 °C to form a presintering mixture powder. To this powder, 0.1 wt.% of Bi_2_O_3_ was added as a melting agent. The powder was then presintered at 1100 °C in air for 2 h at a temperature ramp rate of 5 °C/min, and the ball milling steps were repeated. The final slurry was dried at 90 °C, and 8% polyvinyl alcohol was added as a binder for the subsequent pressing and mixing steps. The final powder was sieved through a 60-mesh sieve three times to obtain granulated powder, and the mixture was then pressed to form a fixed-size primary embryo block. The block was placed in a muffle furnace and heated from room temperature to 900 °C at 5 °C/min. After holding at 900 °C for 30 min, the temperature was increased to 1400 °C at 2 °C/min and sintered at 1400 °C in air for 8 h. The prepared sample was a small black cylinder weighing approximately 10 g.

### 2.2. Sample Characterization

A density calculator (MDJ-300 A, Xiongfa, Shenzhen, China) was used to calculate the true density of the sample. The data were read by placing the sample block on a weighing table and a suspension table in water. The sample was crushed within an agate container, and small particles with a mass of ≤20 mg were selected. The surface was cleaned and gold-sprayed, and the sample morphology was observed by scanning electron microscopy (SEM, Regulus 8230, Hitachi, Tokyo, Japan) at 2000×. The crystal structure of the sintered sample was determined by X-ray diffractometry (SmartLab 9 KW, Rikagaku, Tokyo, Japan) at a scanning speed of 5°/min and scanning angle of 20–70°. Small particles with a mass of ≤20 mg were selected, and the M-H hysteresis line of the sample was measured at 26 °C using a Magnetic Properties Measurement System (MPMS3, Quantum Design, San Diego, CA, USA). The sample was polished into smooth beads with a diameter of ~1 mm, and the ferromagnetic resonance linewidth of the sample at 9.37 GHz was measured using a micro-ferromagnetic resonance system (DH811B, Beijing, China, DaHua Radio Instrument) [14].

## 3. Results and Discussion

### 3.1. Phase Analysis

**Figure 1:**
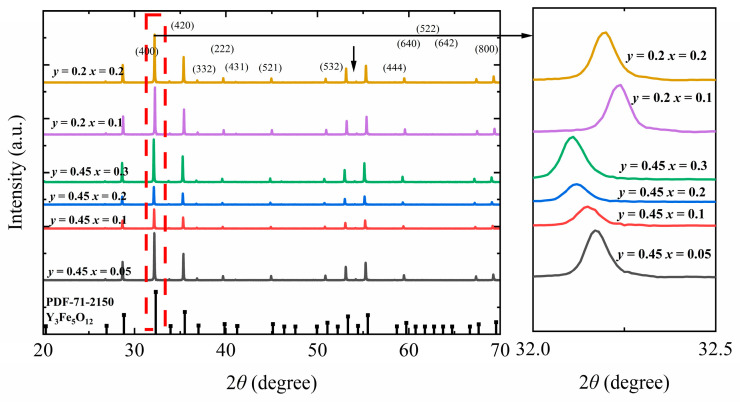
XRD pattern of Y_3−*x*_Ca_x_Fe_5−*x*−*y*_In*_y_*Sn*_x_*O_12_ ferrite.

Figure 1 shows the XRD patterns of Y_3−*x*_Ca*_x_*Fe_5−*x*−*y*_In*_y_*Sn*_x_*O_12_. The *x* range (for Ca^2+^ and Sn^4+^) is 0.05–0.3, whereas the *y* values (for In^3+^) are 0.2 and 0.45. In all samples, only a single YIG phase was formed, consistent with PDF#71-2150. Upon varying the doping concentrations of Ca^2+^ and Sn^4+^ from *x* = 0.05 to 0.3 and maintaining a fixed In^3+^ doping ratio at *y* = 0.45, the main diffraction peak (420) in the obtained pattern shifts from 32.18° to 32.10°. Conversely, upon changing the In^3+^ doping ratio from 0.2 to 0.45 and maintaining a fixed Ca^2+^ and Sn^4+^ doping ratio of 0.2, the main diffraction peak (420) in the obtained pattern shifts from 32.22° to 32.12°. The raw XRD profiles were fitted using Jade 6.5 to determine the lattice constants (Figure 1) [15,16].

**Figure 2:**
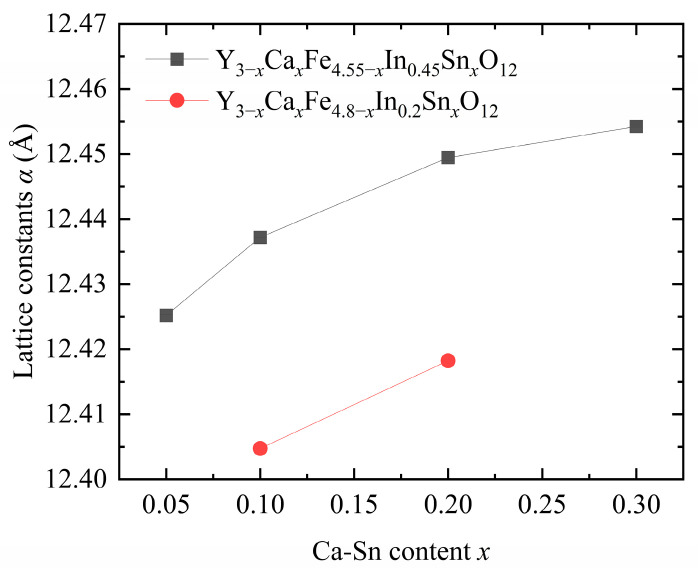
Lattice constants of Y_3−*x*_Ca*_x_*Fe_5−*x*−*y*_In*_y_*Sn*_x_*O_12_.

Figure 2 shows that the lattice constants of the samples gradually increased with increasing In^3+^ and Ca^2+^–Sn^4+^ contents [7,9,12]. The ionic radii of the introduced Ca^2+^ (1.120 Å), In^3+^ (0.790 Å), and Sn^4+^ (0.690 Å) ions were larger than those of Fe^3+^ (0.645 Å) and Y^3+^ (1.015 Å), leading to an increase in the overall size and deformation of the lattice. In addition, the introduction of In^3+^ ions significantly affects the lattice constant [17], consistent with the trend of lattice constants obtained upon pure-phase doping.

### 3.2. Microstructure and Density

**Figure 3:**
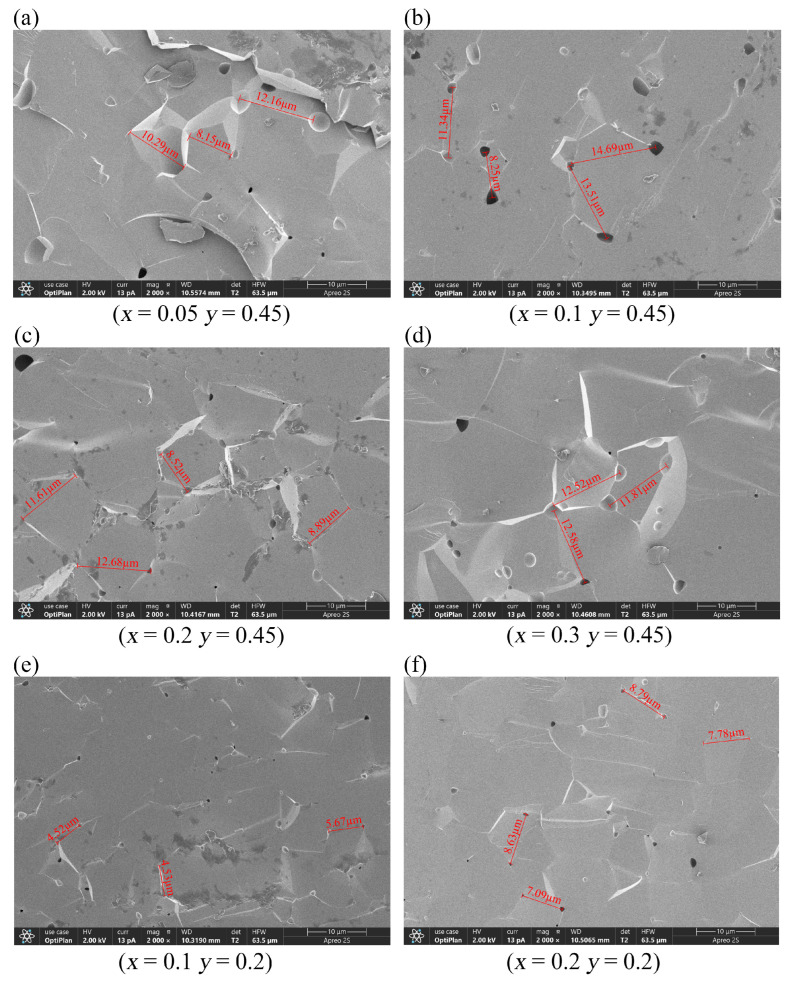
SEM images of (**a**–**d**) Y_3−*x*_Ca*_x_*Fe_4.55−*x*_In_0.45_Sn*_x_*O_12_ (*x* = 0.05–0.3) and (**e**,**f**) Y_3−*x*_Ca*_x_*Fe_4.8−*x*_In_0.2_Sn*_x_*O_12_ (*x* = 0.1, 0.2).

Figure 3 shows the grain morphologies of the samples observed through SEM. D80 denotes the average of most grain sizes, reflecting material uniformity, whereas D100 corresponds to the largest grain size observed, allowing the identification of abnormally large grains. Our samples showed large grain sizes with extremely thin grain boundaries; therefore, grain distribution is not clearly visible under an electron microscope. This result suggests that the samples have extremely high densities, which are conducive to various applications. Y_2.9_Ca_0.1_Fe_4.45_In_0.45_Sn_0.1_O_12_ and Y_2.8_Ca_0.2_Fe_4.35_In_0.45_Sn_0.2_O_12_ exhibited the smallest pores and grain sizes, indicating that highly compact samples can be obtained under the sintering conditions employed in this study. Although all samples were sintered at 1400 °C, their optimal sintering temperatures were inversely proportional to the doping concentration; that is, as the doping concentration increased, the optimal sintering temperature decreased. The sintering temperature reportedly significantly affects crystal size and homogeneity [18].

The theoretical density is calculated using Equation (1) [19]:

(1) $$\rho =\frac{ZW_{m}}{N_{A}a^{3}},$$

where $N_{A}$ = 6.02 × 10^23^ is Avogadro’s constant, *a* is the lattice constant obtained from XRD data (specific data are presented in Table 1 and Table 2), Z is the number of ions per unit cell, the number of YIG molecules is fixed at eight, and $W_{m}$ is the molecular weight of the samples determined from their individual chemical compositions (formulas). The actual densities of the samples were measured using a density-testing apparatus. Equation (2) is used to calculate the porosity of the samples [9]:

(2) $$P=\left(1-\frac{\rho_{0}}{\rho }\right)\times 100\%,$$

where $\rho_{0}$ and ρ denote the apparent and theoretical densities, respectively.

The Ca^2+^, In^3+^, and Sn^4+^ ions introduced into YIG increase its lattice constant and molecular weight [7,9]. At a sintering temperature of 1400 °C, the actual density of Y_3−*x*_Ca*_x_*Fe_4.55−*x*_In_0.45_Sn*_x_*O_12_ is >5.2 g/cm^3^, which indicates that regulating the concentration of doped In^3+^ is conducive to synthesizing high-density garnet ferrites with large lattice constants [7,12].

In the case of the Y_3−*x*_Ca*_x_*Fe_4.55−*x*_In_0.45_Sn*_x_*O_12_ (*x* = 0.1) sample, the porosity was minimized because the sample was sintered at a uniform temperature, and when *x* = 0.1 and *x* = 0.2, the optimal sintering temperature of the corresponding sample was ~1400 °C. An excessively low sintering temperature leads to an incomplete reaction, and unreacted oxides and intermediate perovskite products remain in the sample. In contrast, sintering at an extremely high temperature leads to excessive grain growth and shrinkage, and the porosity increases rather than decreases, which is unfavorable for improving the properties of ferrites [12]. Therefore, although the Ca^2+^–Sn^4+^ doping of YIG lowers the sintering temperature and increases its compactness, the porosity does not always decrease. The actual densities of Y_3−*x*_Ca*_x_*Fe_4.55−*x*_In_0.45_Sn*_x_*O_12_ and Y_3−*x*_Ca*_x_*Fe_4.8−*x*_In_0.2_Sn*_x_*O_12_ were limited by the effects of sintering and the number of ions introduced. In addition, while In^3+^ doping increases the theoretical and actual densities [7], the actual density does not increase linearly with the theoretical density. These observations indicate that the porosity of the samples is collectively influenced by the parameters used in the ferrite preparation.

### 3.3. Magnetic Properties

**Figure 4:**
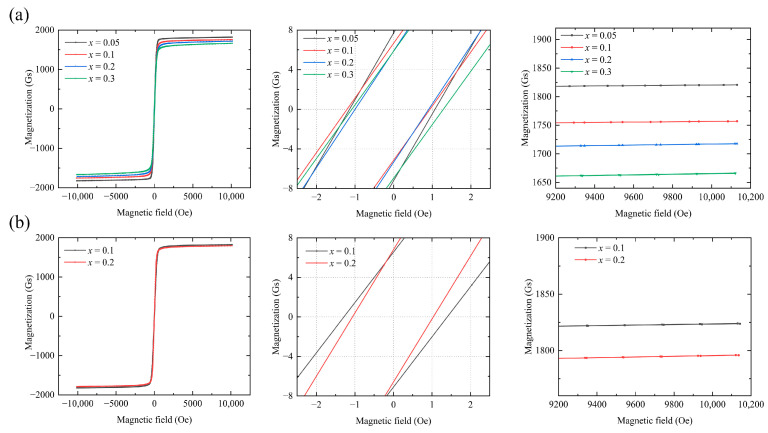
Hysteresis curves of (**a**) Y_3−*x*_Ca*_x_*Fe_4.55−*x*_In_0.45_Sn*_x_*O_12_ and (**b**) Y_3−*x*_Ca*_x_*Fe_4.8−*x*_In_0.2_Sn*_x_*O_12_.

Figure 4 shows the hysteresis curve of Y_3−*x*_Ca*_x_*Fe_5−*x*−*y*_In*_y_*Sn*_x_*O_12_. The M-H hysteresis line of the sample was measured at 26 °C. As a soft magnetic material, the garnet ferrite exhibits a fast magnetization response and an overall narrow hysteresis curve. Once the curve stabilizes, the saturation magnetization ($M_{s}$) decreases as *x* increases.

At 26 °C (where the saturation value of the theoretical magnetization strength of the soft magnetic material cannot be achieved by merely increasing the magnetic field strength H), the actual saturation magnetization is estimated from the M–H curve of the material. When the applied magnetic field H is relatively large, the magnetization strength tends to saturate. In this state, the polycrystalline magnetization curve can be described by the law of approach to saturation [12,20]:

(3) $$M=M_{s}\left(1-\frac{a}{H}-\frac{b}{H^{2}}\right)+\chi_{p}H,$$

where *a* is the magnetic hardness coefficient, *b* is the constant related to the rotation process of the magnetization vector, M_s_ is the saturation magnetization, $\chi_{p}$ is the paramagnetization rate, and $\chi_{p}H$ is the effect of the paramagnetization process on the magnetization under extremely strong magnetic fields and therefore can be neglected. Equation (3) can be simplified as

(4) $$M=M_{s}\left(1-\frac{a}{H}-\frac{b}{H^{2}}\right).$$

The actual saturation magnetization is calculated from data in the strong-magnetic-field region of the M–H curve. H^−2^ in Equation (4) is small and can be neglected. Within the range of strong magnetic fields, M approaches M_s_; thus, we obtain the following [20]:

(5) $$M=M_{s}\left(1-\frac{a}{H}\right),\;\frac{M_{s}a}{H}=M_{s}-M,\;\frac{M}{H}=\frac{M}{M_{s}}\frac{M_{s}-M}{a}\;=\;\frac{M_{s}}{a}\;-\;\frac{M}{a}$$

**Figure 5:**
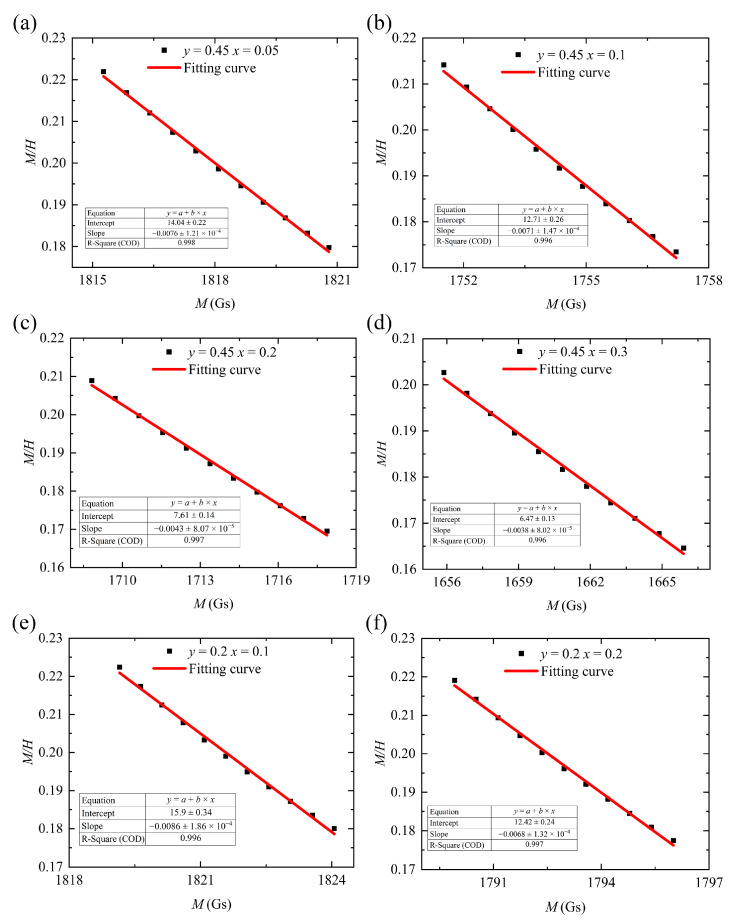
M/H and M fitting curves for (**a**–**d**) Y_3−_*_x_*Ca*_x_*Fe_4.55−_*_x_*In_0.45_Sn*_x_*O_12_ and (**e**,**f**) Y_3−_*_x_*Ca*_x_*Fe_4.8−_*_x_*In_0.2_Sn*_x_*O_12_.

We plotted M on the horizontal axis and $\frac{M}{H}$ on the vertical axis. Data points where M exceeds 1700 Gs (the strong-magnetic-field region) were fitted to a straight line (Equation (5)). This allowed us to directly obtain the slope $\frac{1}{a}$ and $\frac{M_{s}}{a}$ data from this line. Finally, by calculating the intersection point of this curve with the horizontal axis, we derived the theoretical saturation magnetization of the sample [20]. The fitting results are presented in Table 3 and Figure 5.

**Figure 6:**
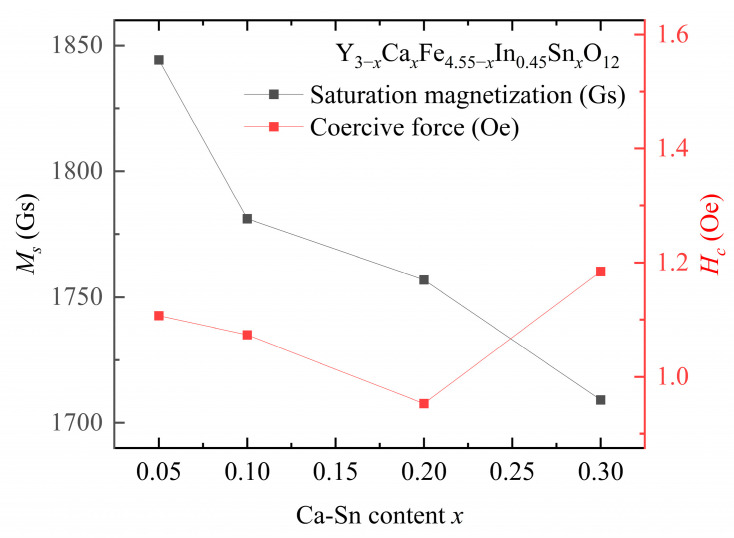
Saturation magnetization ($M_{s}$) and coercive force ($H_{c}$) of Y_3−*x*_Ca*_x_*Fe_4.55−*x*_In_0.45_Sn*_x_*O_12_.

The saturation magnetization and coercive force are presented in Figure 6 and Table 4 and Table 5, respectively.

At a fixed In^3+^ doping concentration, the coercive force of the samples first decreases and then increases with increasing Sn^4+^ content. The lowest coercive force of the sample, i.e., $H_{c}$ = 0.95 Oe, is observed at *y* = 0.45, *x* = 0.2. The coercive force shown by the Ca–In–Sn YIG samples is lower than that of other ion-substituted YIG samples [7,15]. Moreover, the coercive force is positively correlated with grain size, and the correlation between coercivity and grain size reported in the present study is consistent with that in a previous study [12].

The magnetocrystalline anisotropy energy predominantly hinders magnetic moment rotation. The stress impediment model states that the decrease in coercive force is associated with the decrease in anisotropy due to the introduction of In^3+^ and Sn^4+^ [21]. Owing to the density reduction caused by the mismatch in the sintering temperature, the coercivity of the sample increased at *x* = 3 instead of decreasing; this trend was verified by the determined anisotropy constant. The coercive force is influenced by the magnetic anisotropy, saturation magnetization, impurity concentrations, porosity, stress, and other defects of the material, which depend on the synthesis conditions of the material [22].

The saturation magnetization values of the samples decreased with increasing In^3+^ and Sn^4+^ contents. Previous studies have indicated that as the In^3+^ content of a sample increases, the saturation magnetization increases at low temperatures, whereas it decreases at room temperature [11]. This phenomenon explains the results obtained in our study: increasing the In^3+^ dopant content from *y* = 0.2 to *y* = 0.45 at a fixed Ca^2+^–Sn^4+^ content reduces the saturation magnetization at room temperature.

The saturation magnetization of YIG mainly stems from the exchange of a–d sites [11,23,24]. The directions of the magnetic moments of the 16a and 24d ions were opposite, whereas those of the magnetic moments of the 24c and 24d ions were identical. The magnetization intensity is calculated as the difference in magnetic moment generated by the amount of Fe^3+^ remaining at the a and d sites in the crystal after the introduction of *α* and *β* amounts of nonmagnetic ions, respectively, as shown in Equation (6):

(6) $$M\;=\;\left|M_{d}-M_{a}-M_{c}\left|\;=\;\right|\left(3-\beta \right)M_{Fe^{3+}}-\left(2-\alpha \right)M_{Fe^{3+}}-M_{c}\right|$$

The substituted In^3+^ and Sn^4+^ ions preferentially occupy the octahedral sites (a site); consequently, the concentration of Fe^3+^ in these octahedral sites decreases, while the total net magnetic moment increases. This result is consistent with the observed increase in saturation magnetization upon In^3+^ and Sn^4+^ doping [7,25]. Upon further increasing the In^3+^ and Sn^4+^ contents, the saturation magnetization of the samples first increases and then decreases [10,12].

These results are obtained primarily because, as the concentration of substituted ions increases, these ions, which initially only substitute the Fe^3+^ ions at the octahedral a-site, gradually substitute those at both the octahedral a- and tetrahedral d-sites. Subsequently, once the introduced amounts of Sn^4+^ and In^3+^ exceed a certain threshold, these ions will tend to enter the tetrahedral d sites [12]. According to Equation (6), this phenomenon reduces the saturation magnetization. The inflection point of this change in ion-substitution behavior occurs at *x* ≈ 0.3 [12], indicating that the decrease in the saturation magnetization of the sample is reasonable after the total substituted amounts of In^3+^ and Sn^4+^ exceed 0.3. This substitution behavior of the codoped In^3+^ and Sn^4+^ ions is consistent with the substitution rule applicable to both ions. [8]. Consequently, as shown in Figure 6, the saturation magnetization value decreases with increasing In^3+^ and Ca^2+^–Sn^4+^ contents.

### 3.4. Anisotropy Constant

In this study, we observed that when polycrystalline magnetic materials approach saturation during magnetization under strong external magnetic fields, domain wall motion ceases, and magnetic moment rotation dominates the magnetization process [12]. Upon further increasing the external field strength, wide-range magnetocrystalline anisotropy occurs, and with a subsequent increase in the field strength, the effect of defects on rotation diminishes, and eventually, the paramagnetization state dominates at excessively high external magnetic field intensities. The empirical equation, shown as Equation (3), reflecting the near-saturation stage of magnetization, can be solved by differentiation using the Origin software (Version Number: Originpro 2024 10.1.0.178) [12,20]:

(7) $$\frac{dM}{dH}\;=\;M_{s}\left(\frac{a}{H^{2}}+\frac{2b}{H^{3}}\right)+\chi_{p}\;=\;M_{s}\left(aH+2b\right)\frac{1}{H^{3}}+\chi_{p},$$

where $\chi_{p}$ is the paramagnetization rate. The data required for the calculation of the anisotropy constant corresponds to magnetic field intensity H of at least 1000 Oe. Under these conditions, H is sufficiently low such that in the fitting equation 2b >> aH, and Equation (7) simplifies to

(8) $$\frac{dM}{dH}\approx \frac{2bM_{s}}{H^{3}}+\chi_{p}.$$

**Figure 7:**
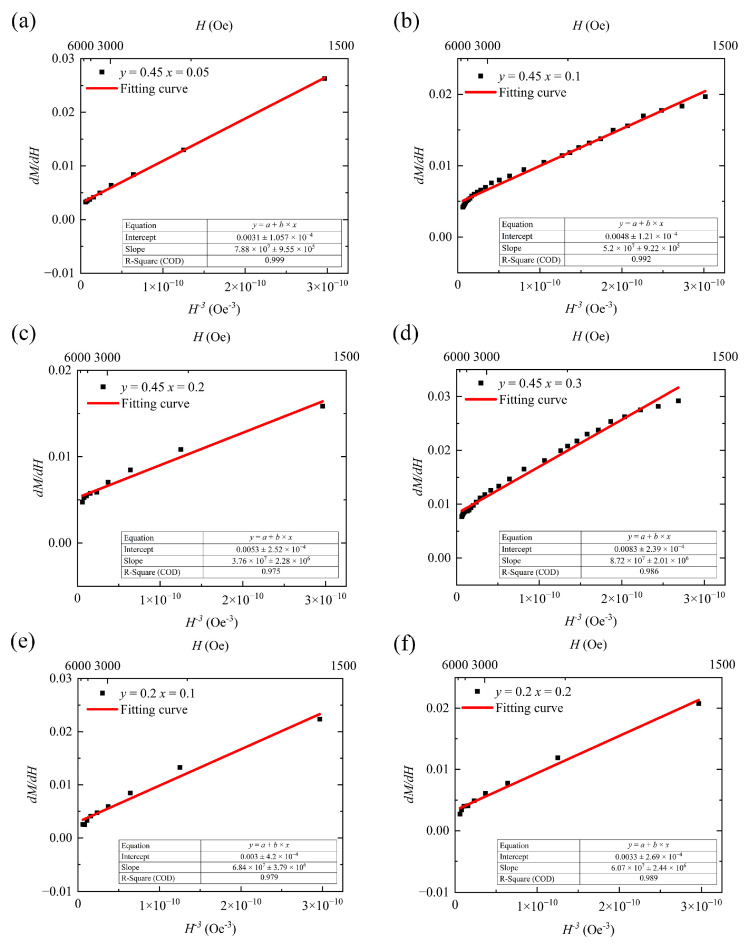
dM/dH and H^−3^ fitting curves for (**a**–**d**) Y_3−*x*_Ca*_x_*Fe_4.55−*x*_In_0.45_Sn*_x_*O_12_ and (**e**,**f**) Y_3−*x*_Ca*_x_*Fe_4.8−*x*_In_0.2_Sn*_x_*O_12_.

The functional relationship between differential susceptibility $\frac{\mathrm{dM}}{\mathrm{dH}}$ and $H^{-3}$ is approximately a straight line near the origin. Applying $\frac{\mathrm{dM}}{\mathrm{dH}}-H^{-3}$ in Equation (8) for curve fitting yields 2bM_s_ as the slope of the fitted straight line, with b reflecting the inhibiting effect of magnetocrystalline anisotropy on domain-reversal magnetization under a strong magnetic field. Equation (9) demonstrates the relationship between the coefficient *b* and the magnetocrystalline anisotropy constant $\left|K_{1}\right|$:

(9) $$b\;=\;\frac{8}{105}\frac{K_{1}^{2}}{\mu_{0}^{2}M_{s}^{2}},$$

where *μ*_0_ is a constant term called the vacuum permeability, and its value is 4π × 10^−7^. The overall fitted curve shown in Figure 7 indicates that within the applied outer field range from 1500 Oe to the maximum value (6000 Oe), the fitted curve well approximates the trend of the measured values, with <3% error. To ensure the reliability of the fitted data, all samples were selected based on the same H values. We have included the H ranges corresponding to the data points in the fitted curve in Figure 7 for reference. When calculating using Equations (8) and (9), the units of M and H are converted to CGS units of Gs and Oe; the coefficient b, obtained from the fitted slope, has units of Oe^3^/Gs; and the unit of the anisotropy constant is kJ/m^3^.

**Figure 8:**
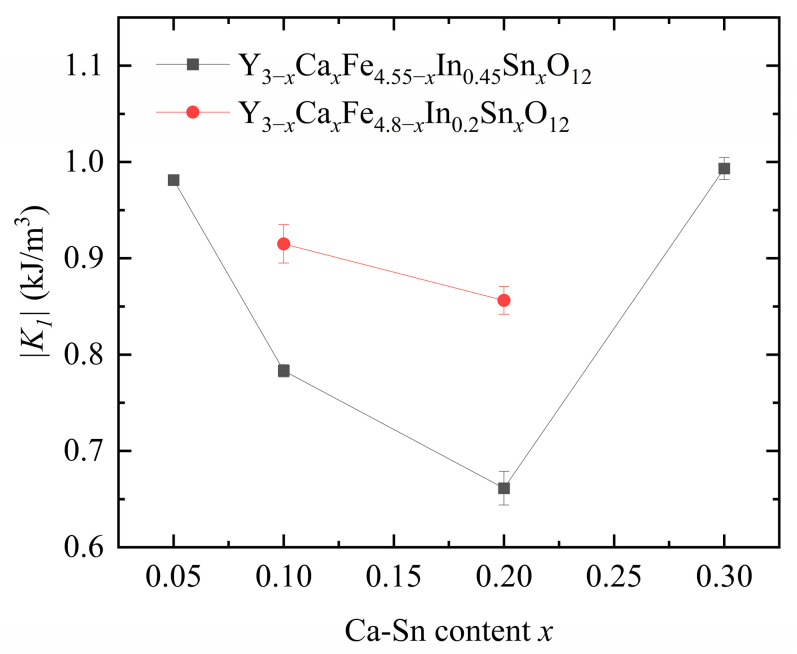
Anisotropy constants of Y_3−*x*_Ca*_x_*Fe_4.55−*x*_In_0.45_Sn*_x_*O_12_ and Y_3−*x*_Ca*_x_*Fe_4.8−*x*_In_0.2_Sn*_x_*O_12_.

Figure 8 displays the calculated anisotropy constants. As shown in Figure 6 above, as the *x* increases, H_c_ first decreases and then increases. This trend is consistent with the calculated anisotropy constant. We observed that the anisotropy constant of our samples decreased significantly with increasing In^3+^ content, indicating a strong effect of In^3+^ substitution on the anisotropy constant.

Upon increasing the Ca^2+^–Sn^4+^ doping concentration at a fixed In^3+^ content, the anisotropy constant first decreased and then increased owing to the change in the position of Fe^3+^ in YIG caused by the substituted Sn^4+^ and In^3+^ ions. The nonmagnetic Sn^4+^ and In^3+^ ions preferentially enter the a-sites in YIG when their concentration is low and begin entering the d-sites as their concentration increases.

Because of the a–d coupling effect, the substitution of Sn^4+^ ions at the a-sites reduces the anisotropy constant, whereas their substitution at the d-sites increases it [21]. The introduction of In^3+^ decreases the anisotropy constant of magnetocrystalline materials [12]. The total dopant amounts of In^3+^ and Sn^4+^ in each of the six samples exceed the turning point of In^3+^ and Sn^4+^ ion substitution from position a to position d (the total amount of the substituted ions is >0.3). The Sn^4+^ ionic radius is larger than that of In^3+^, indicating that Sn^4+^ substitution is favored over that of In^3+^ at the tetrahedral d sites.

Therefore, when the In^3+^ content is controlled at 0.2 and 0.45, the introduced Sn^4+^ ions are more likely to occupy the d-sites, and In is partially substituted at the a- and d-sites; this phenomenon eventually leads to an increase in the anisotropy constant. Therefore, when the amounts of Sn^4+^ and In^3+^ substituted in YIG are low, the substitution rules of these ions for YIG are similar. However, as the substitution amount increases, the rules governing Sn^4+^ and In^3+^ substitution in YIG differ, and the substitution amounts and trends of these ions show an interdependence [6].

### 3.5. Ferromagnetic Resonance Linewidth (FMR)

Because the samples prepared and investigated in this study are polycrystalline, other factors that influence single-crystal properties were also analyzed. An analysis of the ferromagnetic relaxation process revealed that the stray field around the pores and anisotropic fluctuations caused by disordered crystal orientations primarily cause the inhomogeneity of the two-magnon scattering field [26,27,28]. The ferromagnetic resonance linewidth of the polycrystalline garnet ferrite is equivalent to the sum of the following linewidth effects:

(10) $$\Delta H\approx \Delta H_{i}+\Delta H_{p}+\Delta H_{a}+\Delta H_{s},$$

where ${\Delta H}_{i}$ is the garnet ferrite single-crystal linewidth; its value is 1 Oe, which only negligibly influences the properties of the garnet ferrite. Because our sample was not doped with rare-earth ions (characterized by strong spin–lattice coupling effects), ${\Delta H}_{i}$ was negligible in this study. In addition, to reduce the measurement error in ${\Delta H}_{s}$, which is affected by the roughness of the polycrystalline surface, the tested samples were subjected to a surface polishing treatment. Prior to measuring the experimental linewidth of the sample, its surface was smoothed and polished via machining; thus, ${\Delta H}_{s}$ was negligible in our case. Therefore, for our samples, Equation (10) can be simplified to

(11) $$\Delta H\approx \Delta H_{p}+\Delta H_{a}.$$

${\Delta H}_{p}$ reflects the effect of the ferromagnetic resonance linewidth caused by the pores in the polycrystalline garnet ferrite. Under an applied magnetic field, the pores inside the sample and nonmagnetic alternative phase surfaces can appear as magnetic charges generating a localized demagnetization field, which increases ${\Delta H}_{p}$ [29,30]. To calculate the magnetization rate of the direct-current component of the additional field arising from nonmagnetic impurities and free magnetic poles on the surface of the pores, all pores in the sample were equated to a spherical pore located at the center of the pore surface. We averaged the weights of the magnetization rates to account for the fluctuations in the demagnetizing field around the pores or other nonmagnetic alternative phases, which are the sources of coupling between the coherent feed and spin wave. This approach was used to derive the pore-induced broadening equation (CGS system) [12]:

(12) $$\Delta H_{p}\;=\;1.5\left(4\pi M_{s}\right)P.$$

**Figure 9:**
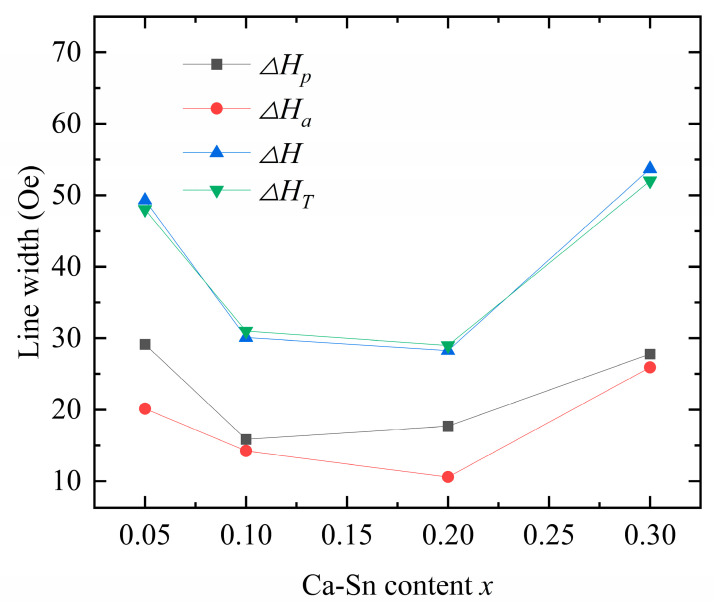
Calculated ${\Delta H}_{a}$,${\;\Delta H}_{p}$, and ΔH values and test results of${\;\Delta H}_{T}$ for Y_3−*x*_Ca*_x_*Fe_4.55−*x*_In_0.45_Sn*_x_*O_12_.

${\Delta H}_{a}$ originates from fluctuations in the anisotropic field ($H_{a}$), arising from the random distribution of magnetocrystalline anisotropy fields among individual grains. Magnetic crystal anisotropy induces direction-dependent equivalent fields along different crystal axes, causing anisotropic dissipation of magnetic moment precession energy. This broadens the resonance peak, with ${\Delta H}_{a}$ specifically reflecting this phenomenon [12,31,32]. When the angular frequency of the applied external magnetic field is the same as the frequency of the electron precession in the sample, the resonant magnetic field exhibits dispersion. The degree of broadening is directly correlated with the anisotropic magnetocrystalline field. Moreover, a magnetic coupling interaction occurs between the grains, and the magnetic distances are parallel, that is, synchronous precession occurs in this system. This effect becomes noticeable at $M_{s}\gg H_{a}$, which indicates the narrowing of ΔH due to the magnetic coupling interaction. At this $M_{s}\gg H_{a}$, ${\Delta H}_{a}$ is expressed as

(13) $$\Delta H_{a}=2.07\frac{{\left(\frac{K_{1}}{\mu_{0}M_{s}}\right)}^{2}}{M_{s}}G\left(\frac{\omega }{\omega_{m}}\right),$$

where $G\left(\frac{\omega }{\omega_{m}}\right)$ is a factor related to the frequency of the external field, and at a frequency of 9.25 GHz, G ≈ 1. The actual ferromagnetic resonance linewidth (${\Delta H}_{T}$) of the samples was measured using a microwave ferromagnetic resonance system. The derived ${\Delta H}_{p}$ and ${\Delta H}_{a}$ values are used to calculate the ferromagnetic resonance linewidth (ΔH) of the samples, and the results are compared with ${\Delta H}_{T}$ (Figure 9; Table 6 and Table 7).

The theoretically calculated values are consistent with the experimental data, underscoring the dual effects of the anisotropic and porosity linewidths as the origin of the ferromagnetic resonance linewidth caused by the Ca–In–Sn doping. Upon increasing the concentration of Sn^4+^ while maintaining a fixed In^3+^ doping concentration, both ${\Delta H}_{p}$ and ${\Delta H}_{a}$ first decreased and then increased. Because porosity is affected by variations in grain size and sintering temperature [18], we can conclude that both excessive and insufficient sintering increase the porosity.

According to Equation (13), the anisotropic linewidth is a function of the anisotropy constant and saturation magnetization; that is, it is proportional to the anisotropy constant and inversely proportional to the saturation magnetization. Our results show that both In^3+^ and Sn^4+^ reduce the ferromagnetic resonance linewidth [33], whereas the introduction of In^3+^ drastically reduces the anisotropy constant. However, when the saturation magnetization of the samples decreased, the observed decreasing trend in the ferromagnetic resonance linewidth was disrupted. In the case of Ca^2+^–Sn^4+^ substitution (*x* = 0.3), the anisotropy of the samples initially increased, leading to an increase in the anisotropic linewidth. Y_2.8_Ca_0.2_Fe_4.35_In_0.45_Sn_0.2_O_12_ exhibits the smallest measured ferromagnetic resonance linewidth of ΔH = 29 Oe, showing the potential for application in various fields.

**Figure 10:**
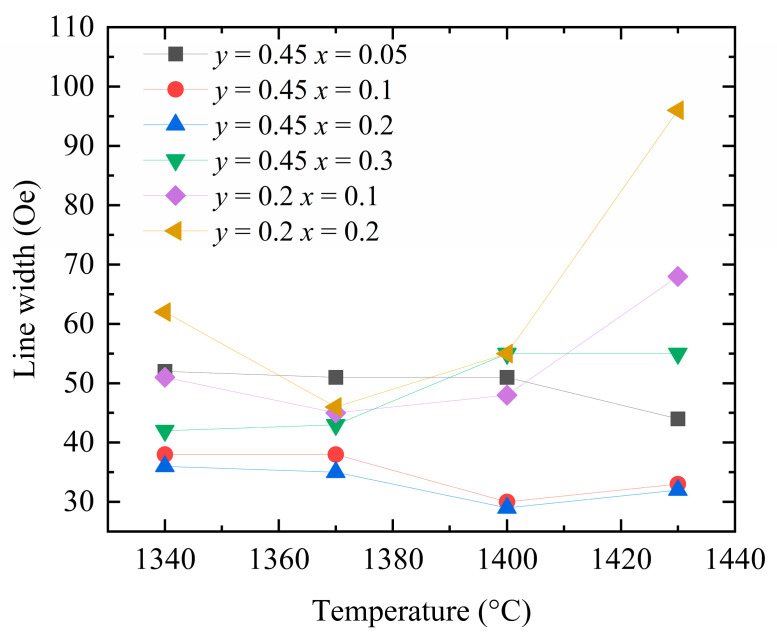
Ferromagnetic resonance linewidth ${\Delta H}_{T}$ of Y_3−*x*_Ca*_x_*Fe_5−*x*−*y*_In*_y_*Sn*_x_*O_12_ at different preparation temperatures.

Figure 10 shows the measured ferromagnetic resonance linewidths of the Y_3−*x*_Ca*_x_*Fe_5−*x*−*y*_In*_y_*Sn*_x_*O_12_ samples sintered at different temperatures. With increasing concentrations of Ca^2+^, In^3+^, and Sn^4+^ dopants, the temperature corresponding to the smallest ferromagnetic resonance linewidth of each sample tends to decrease. This observation indirectly indicates that the introduction of Ca–In–Sn results in a decrease in the required sintering temperature. Upon lowering the sintering temperature to 1360–1380 °C, the ferromagnetic resonance linewidths of the two groups of the samples doped with Ca^2+^–Sn^4+^ (*x* = 0.2 and 0.3) decrease further, while the samples become more compact. These results demonstrate that the observed changes in the ferromagnetic resonance linewidth of the YIG samples with the sintering temperature stem from the porosity linewidth.

## 4. Conclusions

In this study, we successfully synthesized YIG ferrites doped with Ca^2+^, In^3+^, and Sn^4+^ using a solid-phase reaction method and investigated the magnetic properties of the ferromagnetic resonance linewidth of Y_3−*x*_Ca*_x_*Fe_5−*x*−*y*_In*_y_*Sn*_x_*O_12_ and the mechanism underlying the linewidth variations. The substitution of In^3+^ and Sn^4+^ usually occurred at the octahedral a-sites; however, when the ionic codopant concentration was >0.3, the saturation magnetization reduced, suggesting a shift in the tendency of In^3+^ and Sn^4+^ to substitute Fe^3+^. The measured $H_{c}$ and $M_{s}$ values confirmed that substituting appropriate amounts of In^3+^ and Sn^4+^ significantly reduced the anisotropy constant. We analyzed various factors that could affect the ferromagnetic resonance linewidth and performed theoretical calculations to determine the mechanism underlying the linewidth variations shown by the samples. Evidently, the ferromagnetic resonance linewidth first decreased and then increased because of dual effects: negative correlation of the linewidth with the saturation magnetization and positive correlation of the same with the anisotropy constant. This result demonstrated that the sample synthesis process plays a crucial role in linewidth variation. Upon sintering at 1400 °C, Y_2.8_Ca_0.2_Fe_4.35_In_0.45_Sn_0.2_O_12_ exhibited the lowest coercive force ($H_{c}$ = 0.95 Oe) and the smallest measured linewidth (ΔH = 29 Oe). These results indicated that elements such as Ca, In, and Sn play a vital role in regulating the ferromagnetic resonance linewidth of YIG. This process minimizes resonance loss and enables efficient sample fabrication and sintering without significantly reducing the saturation magnetization intensity.

## Figures and Tables

**Table 1:** Density, lattice constant (*a*), porosity (P), and grain size of Y_3−*x*_Ca*_x_*Fe_4.55−*x*_In_0.45_Sn*_x_*O_12_.

*x*	Molecular Weight	Lattice Constant a (Å)	Theoretical Density (g/cm^3^)	Actual Density (g/cm^3^)	Porosity (%)	D80 (μm)	D100 (μm)
0.05	765.18	12.425	5.3	5.245	1.054	10	13
0.1	765.88	12.437	5.291	5.258	0.611	9	12
0.2	767.29	12.449	5.284	5.249	0.671	10	13
0.3	768.69	12.463	5.277	5.221	1.084	12	16

**Table 2:** Density, lattice constant (*a*), porosity (P), and grain size of Y_3−*x*_Ca*_x_*Fe_4.8−*x*_In_0.2_Sn*_x_*O_12_.

*x*	Molecular Weight	Lattice Constant a (Å)	Theoretical Density (g/cm^3^)	Actual Density (g/cm^3^)	Porosity (%)	D80 (μm)	D100 (μm)
0.1	751.14	12.405	5.229	5.175	1.04	5	7
0.2	752.54	12.418	5.222	5.16	1.189	7	8

**Table 3:** $M-\frac{M}{H}$ fitted data for Y_3−_*_x_*Ca*_x_*Fe_5−_*_x_*_−_*_y_*In*_y_*Sn*_x_*O_12_: Slope $\frac{1}{a}$, Constant $\frac{M_{s}}{a}$, Fitting Error Summary.

*y*	*x*	$\frac{M_{s}}{a}$	$\frac{1}{a}$	R−Square
0.45	0.05	14.04	0.0076	0.998
0.45	0.1	12.71	0.0071	0.996
0.45	0.2	7.61	0.0043	0.997
0.45	0.3	6.47	0.0038	0.996
0.2	0.1	15.9	0.0086	0.996
0.2	0.2	12.42	0.0068	0.997

**Table 4:** Saturation magnetization ($M_{s}$) and coercive force ($H_{c}$) of Y_2.9_Ca_0.1_Fe_4.9−*y*_In*_y_*Sn_0.1_O_12_ with different doped In^3+^ amounts (*y*).

*y*	$M_{s}$ (Gs)	$H_{c}$ (Oe)
0.2	1844.98	1.34
0.45	1781.11	1.07

**Table 5:** Saturation magnetization ($M_{s}$) and coercive force ($H_{c}$) of Y_2.8_Ca_0.2_Fe_4.8−*y*_In*_y_*Sn_0.2_O_12_ with different doped In^3+^ amounts (*y*).

*y*	$M_{s}$ (Gs)	$H_{c}$ (Oe)
0.2	1822.45	1.05
0.45	1756.87	0.95

**Table 6:** Calculated (ΔH) and measured (${\Delta H}_{T}$) linewidths of Y_2.9_Ca_0.1_Fe_4.9−*y*_In*_y_*Sn_0.1_O_12_ with different In^3+^ doping amounts (*y*).

*y*	$\Delta H_{p}$ (Oe)	$\Delta H_{a}$ (Oe)	ΔH (Oe)	$\Delta H_{T}$ (Oe)
0.2	28.78	17.48	46.26	47
0.45	15.86	14.25	30.11	31

**Table 7:** Calculated (ΔH) and measured (${\Delta H}_{T}$) linewidths of Y_2.8_Ca_0.2_Fe_4.8−*y*_In*_y_*Sn_0.2_O_12_ with different In^3+^ doping amounts (*y*).

*y*	$\Delta H_{p}$ (Oe)	$\Delta H_{a}$ (Oe)	ΔH (Oe)	$\Delta H_{T}$ (Oe)
0.2	32.51	15.88	48.39	49
0.45	17.68	10.59	28.27	29

## Data Availability

The raw data supporting the conclusions of this article will be made available by the authors on request.
